# Development of Colorectal-Targeted Dietary Supplement Tablets Containing Natural Purple Rice Bran Oil as a Colorectal Chemopreventive

**DOI:** 10.3390/nu10040444

**Published:** 2018-04-04

**Authors:** Busaban Sirithunyalug, Chalermpong Saenjum, Suporn Charumanee, Bhagavathi Sundaram Sivamaruthi, Chaiyavat Chaiyasut, Jakkapan Sirithunyalug, Pratchaya Tipduangta

**Affiliations:** 1 Department of Pharmaceutical Sciences, Faculty of Pharmacy, Chiang Mai University, Chiang Mai 50200, Thailand; busaban.s@cmu.ac.th (B.S.); chalermpong.saenjum@gmail.com (C.S.); chsuporn@gmail.com (S.C.); sivasgene@gmail.com (B.S.S.); chaiyavat@gmail.com (C.C.); Jakkapan.S@cmu.ac.th (J.S.); 2 Cluster of Excellence on Biodiversity based Economics and Society (B.BES-CMU), Chiang Mai University, Chiang Mai 50200, Thailand; 3 Innovation Center for Holistic Health, Nutraceuticals and Cosmeceuticals, Faculty of Pharmacy, Chiang Mai University; Chiang Mai 50200, Thailand

**Keywords:** γ-oryzanol, purple rice bran oil, delivery system for dietary supplement, gastrointestinal inflammatory diseases, anti-inflammatory activity

## Abstract

Colorectal cancer occurs due to various factors. The important risks are dietary lifestyle and inflammatory bowel diseases, such as Crohn’s disease and ulcerative colitis. It has been found that the inhibitory enzyme cyclooxygenase-2 (COX-2) in the colorectal region can potentially reduce the risk of colorectal cancer. The present study investigated rice bran oil from natural purple rice bran, which exhibits antioxidant and anti-inflammatory activity. This study aimed to evaluate the bioactive compound content of natural purple rice bran oil (NPRBO) derived from native Thai purple rice and the anti-inflammatory activity of NPRBO in colorectal cancer cells, and to develop a colorectal delivery platform in the form of film-coated tablets. NPRBO from the rice bran of five different Thai purple rice cultivars, namely Khao’ Gam Leum-Phua (KGLP), Khao’ Gam Boung (KGB), Khao’ Gam Thor (KGT), Khao’ Gam Pah E-Kaw (KGPEK), and Khao’ Niaw Dam (KND), were extracted using the supercritical carbon dioxide extraction technique. The amount of γ-oryzanol (ORY), tocotrienols, and tocopherols present in NPRBOs and the in vitro anti-inflammatory activity of NPRBO were investigated. The highest anti-inflammatory NPRBO was transformed into a dry and free-flowing powder by liquisolid techniques. Then, it was compressed into core tablets and coated with Eudragit^®^L100 and Eudragit^®^ NE30D. The in vitro release study of the film-coated NPRBO tablets was performed in three-phase simulated gastrointestinal media. The cultivar KGLP was superior to the other samples in terms of the ORY, tocotrienol and tocopherol contents and anti-inflammatory activity. Aerosil^®^ was the most suitable absorbent for transforming NPRBO into a free-flowing powder and was used to prepare the NPRBO core tablets. The in vitro KGLP-NPRBO film-coated tablet release profile showed that no ORY was released at gastric pH while 85% of ORY was released at pH 7.4 after 6 h; this would be expected to occur in the colorectal area. Therefore, this study demonstrates the potential of KGLP-NPRBO to prevent colorectal cancer via a specific colorectal dietary supplement delivery system.

## 1. Introduction

Colorectal cancer is a type of cancer that develops in the colon or rectum. Symptomatically, patients suffer from bloody stools, weight loss, bowel movement changes (such as diarrhea, constipation, or narrowing of the stool), and fatigue. Colorectal cancer occurs due to various factors. Inadequate fiber intake and inflammatory bowel diseases, such as Crohn’s disease and ulcerative colitis, significantly increase the risk of colorectal cancer [1,2] The occurrence of colorectal cancer is high—over 1 million people worldwide suffer from this disease. The five-year survival rate for colorectal cancer is less than 60% [1,2]. One third of colorectal cancer patients die in developed countries [1]. If colorectal cancer is detected at an early stage, surgery, chemotherapy, and radiation therapy are the treatment choices. It has been reported that using either aspirin or celecoxib, which inhibit cyclooxygenase-2 (COX-2), can reduce the risk of colorectal cancer in high-risk groups, such as obese individuals and those who smoke and lack physical exercise [2,3]. However, the use of aspirin and celecoxib is limited in patients with a high risk of bleeding, gastric ulcers and cardiovascular disease, and is contraindicated in chronic kidney failure patients.

Rice is an important plant for the Thai economy. It is one of the most famous Thai agricultural products and is exported worldwide. Rice bran is a valuable byproduct of the rice milling process. Particularly, it is rich in dietary fibers, γ-oryzanol, essential vitamin E complex, tocotrienols, and β-sitosterol [4]. Several reports have shown that the extracts from rice bran contain biologically active compounds that possess antioxidant, chemopreventive, anti-inflammatory, antimutagenic, and anticarcinogenic properties [5,6,7]. Rice bran oil is obtained from the inner husk and germ of rice. It is a rich source of γ-oryzanol, essential vitamin E complex, tocotrienols, tocopherols, and β-sitosterol [4]. Rice bran oil has various health benefits, including reducing the risk of cardiovascular disease, preventing cancer, lowering blood cholesterol, and inhibiting platelet aggregation in both in vitro and in vivo studies, in addition to its antioxidant and anti-inflammatory activities [8,9,10]. γ-Oryzanol is a bioactive compound in rice bran oil that plays a vital role in conferring these health benefits. The acute toxic dose of γ-oryzanol is greater than 25 g/kg of body weight in rats and mice [11]. However, the consumption of γ-oryzanol at a dose of 2 g/kg of body weight per day for two years has been shown to be safe and non-carcinogenic in rats [12]. Therefore, rice bran oil has been commercialized as a food supplement in many countries.

Thai purple rice is a native plant that is cultivated in the Northern part of Thailand, particularly in the highlands and mountainous areas. The natural purple rice bran oil (NPRBO) obtained from the Khao’ Gam Pah E-Kaw, Khao’ Gam Thor, and Khao’ Gam Boung varieties with an γ-oryzanol content greater than 5% has demonstrated excellent in vitro antioxidant and inhibitory effects on nitric oxide production in mouse macrophages [10]. To the best of our knowledge, no study has intensively investigated the chemopreventive effects of NPRBO in colorectal cancer. We speculate that NPRBO is a potential candidate as a chemopreventive agent for colorectal cancer. One challenge is that NPRBO is in liquid form, meaning that most of it is absorbed in the stomach or duodenum after administration; therefore, γ-oryzanols reach the colorectal region at concentrations insufficient for preventing colorectal cancer.

Colorectal-targeted delivery is an approach that allows the active ingredient to be released in the specific region from the colon to the rectum. This can be achieved by using a polymer with pH-dependent solubility (soluble at pH 6.0–7.0) combined with a time-dependent controlled release polymer to prevent premature release in the gastrointestinal region. This can be achieved with a polymeric matrix containing active ingredient or polymer-coated tablets. This study selected the double coating layer approach, i.e., a pH-dependent soluble polymer, Eudragit^®^L100, as the outer coating layer and a time-controlled release polymer, Eudragit^®^ NE30D, as the inner coating layer of the NPRBO core tablets. The outer coating layer protects the core tablets from dissolving in the stomach, duodenum and early part of the ileum, and the inner coating layer delays NPRBO release until the colorectal area.

Our study aimed to evaluate the bioactive compounds in NPRBO, including γ-oryzanol, tocotrienols, and tocopherols and to investigate the anti-inflammatory activities of NPRBO by inhibiting inducible nitric oxide (iNOS) and COX-2 production in a cell-based study. Additionally, this study provided a proof of concept in vitro dietary supplement delivery system for NPRBO film-coated tablets, with the aim of releasing NPRBO in the colorectal region where it can act as a colorectal cancer chemopreventive agent.

## 2. Material and Methods

### 2.1. Chemicals and Materials

γ-Oryzanol was purchased from Wako Pure Chemical Industries (Japan). δ-, β-, γ-, and α-tocotrienol, and δ-, β-, γ-, and α-tocopherol were purchased from Merck Co., Ltd. (Kenilworth, NJ, USA). All solvents and chemicals used were either HPLC grade or analytical grade and were purchased commercially from Sigma Chemical Co. (St. Louis, MO, USA), Fluka Chemie GmBH(St. Gallen, Switzerland), Merck (Darmstadt, Germany), Invitrogen (Carlsbad, CA, USA), and Roche (Basel, Germany). A murine macrophage cell line (RAW 264.7), a human adenocarcinoma cell line (HT-29), and a colorectal carcinoma cell (HCT 116) were purchased from American Cell Culture Collection (Bethesda, MD, USA). Talcum was purchased from Union Science Co., Ltd. (Chiang Mai, Thailand). Eudragit^®^ NE30D and Eudragit^®^ L100 were supplied by Evonik industries AG (Essen, Germany). Colloidal silicon dioxide (Aerosil^®^) was purchased from Bio-Chemical Technology Co., Ltd. (Prathum Thani, Thailand). Microcrystalline cellulose Avicel^®^ was purchased from Sigma-Aldrich (St. Louis, MO, USA).

### 2.2. Preparation of Natural Purple Rice Bran Oil (NPRBO)

Five local glutinous purple rice cultivars (*Oryza sativa* L.) were collected from Northern Thailand in December 2010. Khao’ Gam Leum-Phua (KGLP), Khao’ Gam Thor and Khao’ Gam Boung were collected from the Phob-Pra Agricultural Extension Office, Tak Province. Khao’ Gam Pah E-Kaw and Khao’ Niaw Dam were collected from the Mae Hong Son Rice Research Center, Mae Hong Son Province. All samples were dried in a hot air oven at 60 °C for 48 h. Then, fresh rice bran samples were obtained from a rice milling process (Thongtawee, Model: NW 1000 TURBO) and sieved through a 60-mesh sieve, then subjected to a heating–cooling process to inactivate endogenous lipase [13]. Then, NPRBOs were prepared by supercritical extraction using carbon dioxide as the solvent at 60 °C with 450 bar of pressure [14].

### 2.3. Determination of the γ-Oryzanol Content 

The γ-oryzanol content of NPRBOs was determined by reversed-phase HPLC, as reported previously using an Agilent model 1200 apparatus (Santa Clara, CA, USA) [14,15]. The 250 × 4.6 mm diameter Symmetry Shield RP18 column was obtained from Waters Co., Ltd. (NSW, Australia). The mobile phase consisted of methanol, acetonitrile, dichloromethane and acetic acid in a ratio of 50:44:3:3. The wavelength of the detector was set at 330 nm, and the flow rate was 1.0 mL/min. All samples were tested in triplicate.

### 2.4. Determination of the Tocotrienol and Tocopherol Content

The δ-, β-, γ-, and α-tocotrienol and δ-, β-, γ-, and α-tocopherol contents in the NPRBOs were optimized according to the improved method of Pengkumsri et al. [16] with slight modifications. Reversed-phase HPLC was performed using Agilent 1200 with a fluorescence detector; the wavelength of the detector was set at 296 nm excitation and 330 nm emission. The 150 × 4.6 mm diameter KINETEX^®^PFP column was obtained from Phenomenex Co., Ltd. (Torrance, CA, USA). The mobile phase consisted of methanol and de-ionized water in a ratio of 9:1. The flow rate was set at 0.6 mL/min. All samples were analyzed in triplicate.

### 2.5. Determination of Anti-Inflammatory Activities

The anti-inflammatory activities of NPRBOs were investigated through inhibitory effects on nitric oxide and iNOS production in RAW 264.7 cells. Moreover, the inhibitory effect of COX-2 production in HCT 116 and HT-29 cells was also investigated. The passage number of RAW 264.7 mouse macrophage and colorectal HCT116 and HT-29 for anti-inflammatory activity determination was between 5–10.

### 2.6. Determination of Nitric Oxide and iNOS Production

The inhibition of nitric oxide and iNOS production was assayed using the improved methods of Hong et al. [17], Hu et al. [18], and Tuntipopipat et al. [19] with some modifications. Initially, RAW 264.7 cells were cultured in Dulbecco’s Modified Eagle’s Medium (DMEM), supplemented with 10% fetal bovine serum (FBS), 100 units/mL penicillin and 100 µg/mL streptomycin. RAW 264.7 cells were used to determine the inhibitory activity on nitric oxide and iNOS production. The cells were pre-incubated in 24-well plates for 24 h. Then, cells were given fresh medium containing various concentrations of the tested samples (10, 25, 50, 75, and100 µg/mL). After 12 h of incubation, lipopolysaccharide (LPS) and interferon-γ (IFN-γ) were added. After 72 h of incubation, the culture medium supernatants were collected to analyze for nitric oxide content, and the cells were lysed to yield cell lysates using CelLytic^TM^ M Cell Lysis Buffer (Sigma, C2978) to perform the assay for iNOS. The quantity of nitrite in the culture medium was measured using Griess reagent as an indicator of nitric oxide production. Finally, the absorbance was measured at 540 nm against a standard curve of potassium nitrite [20]. Fresh culture medium was used as a blank. Moreover, a commercially available mouse iNOS ELISA kit (CSB-E08326M, Cusabio Biotech, Co., Ltd., Houston, TX, USA) was used to measure the production of iNOS in the cell lysates. Curcumin and γ-oryzanol were used as the positive controls. The amount of DNA was quantified by the Quant-iT PicoGreen Assay (Invitrogen, P11496) in accordance with to the manufacturer’s protocol, while that of the protein produced by RAW 264.7 cells was analyzed using Bradford reagent prepared in-house [21]. Concurrently, the viability of RAW 264.7 cells was assayed according to the improved methods of Jomha et al. [22] and Saenjum et al. [10] with slight modifications. The effect of NPRBOs on cell viability was assayed after stimulation with LPS and IFN-γ in the absence or presence of NPRBOs for 72 h using the cell proliferation reagent WST-1 (Roche, Basel, Switzerland).

### 2.7. Determination of COX-2 Production

The inhibition of COX-2 production was assayed using a human total COX-2 immunoassay (RayBiotech, Inc., Norcross, GA, USA) following the methods of Hong et al. [17] and Colucci et al. [23] with slight modifications. To determine the inhibitory effects on COX-2 production, HT-29 and HCT116 cells (1 × 10^5^ cells/well) were pre-incubated in a black 96-well plate with a clear bottom and incubated for 24 h. Then, cells were given fresh medium containing various concentrations of the tested samples (final concentration 10–100 µg/mL). After 12 h of incubation, LPS and IFN-γ were added. After 72 h of incubation, cell supernatants, which were extracted with 10 mM Tris, pH 8.0, 1% NP-40, 0.15 M NaCl, and 1 mM EDTA were collected to analyze total COX-2 levels. The procedure to determine the level of production followed the manufacturer’s protocol. Curcumin and γ-oryzanol were used as the positive controls. Concurrently, the viability of HT-29 and HCT116 cells were assayed using the cell proliferation reagent, WST-1. The amount of DNA was quantified by the Quant-iT PicoGreen Assay (Invitrogen, P11496), in accordance with the manufacturer’s protocols, while that of the protein produced by HT-29 and HCT116 cells was analyzed using Bradford reagent prepared in-house [21].

### 2.8. Preparation of NPRBO Core Tablets

NPRBO was converted into a dry powder using absorbents, including microcrystalline cellulose (Avicel^®^ pH 101) and colloidal silicon dioxide (Aerosil^®^). Then, the binding solution, 10% PVP-K 90 in isopropanol, was added while continuously mixing until a wet mass was obtained. The damp mass was sieved through No. 8 mesh sieve, and the NPRBO granules were dried at room temperature overnight, followed by another sieving through No. 12 mesh sieve. Then, they were mixed with tableting excipients, including talcum (1%), Ac-Di-Sol^®^ (0.5%), and magnesium stearate (0.5%), by tumbling the mixture in a polypropylene bag for 2 min. A B3B rotary tablet compressor (Manestry Machine Limited, Liverpool, UK), equipped with 8.2 mm dies, was used to prepare tablets, with a compression force between 12 and 15 kN. Some tablets were selected to perform tablet quality control tests, including assessing the γ-oryzanol content, weight variation, hardness, and friability. The hardness of the tablet was tested using a Monsanto Tablet Hardness Tester (Monsanto Tablet Hardness Tester, Model: C-MHT 20, Scilution Co. Ltd., Bang Bua Thong, Thailand), and the average values were obtained. Tablet friability was measured using a friability tester (Pharma Test, Hainburg, Germany).

### 2.9. Coating of NPRBO Tablets

Two coating layers, including Eudragit^®^ NE30D (inner coating) and Eudragit^®^ L100 (enteric coating), were coated onto the NPRBO tablets for the purpose of colorectal-targeted delivery.

Two types of coating solution (inner coating and outer coating solution) were used to coat the NPRBO core tablets. The inner layer coating solution contained 10% *w*/*v* Eudragit^®^ NE30D and 25% talcum as the coating material. The outer layer coating solution contained Eudragit^®^ L-100 D and 25% talcum as the coating material. A Thai coater (15PMS Supply Ltd., Bangkok, Thailand) was used to coat the NPRBO core tablets. The rotating rate of the coating pan was 9–11 rpm, the spraying rate of the coating solution was 2.83–3.11 g/min, the air pressure was 2.5–3.5 bar, the temperature in the coating pan was 97–103 °C, and the coating duration was 1.5–6 h, at 17–18% relative humidity. After coating, the tablets were cured at 40 °C for 2 h. Then, some of the coated tablets were used to perform tablet quality control tests as described in the previous section.

### 2.10. In Vitro Gastrointestinal γ-Oryzanol Release Study

A dissolution apparatus II (SR8PLUS, Hanson Virtual) was used to perform the dissolution study at 37 ± 0.5 °C with a paddle speed of 100 rpm. The NPRBO coated tablets were studied regarding the release of γ-oryzanol in a series of media, including 1 N hydrochloric acid, pH 1.2, for 2 h; phosphate buffer, pH 6.8, for 3 h; and phosphate buffer, pH 7.4, for 2 h, to simulate the human gastrointestinal environment. The media were sampled at time points of 120, 300, 310,320, 330, 360, 390 and 420 min. The γ-oryzanol in the media was evaluated by HPLC (Agilent 1200).

### 2.11. Analysis of the γ-Oryzanol Content

The tablets were crushed, extracted with hexane, and then the solvent was evaporated. The extracts were analyzed for their γ-oryzanol content by HPLC, as stated previously.

### 2.12. Statistical Analysis

All of the results are expressed as the mean of three replicates ± standard deviation (SD). All statistical analyses were conducted using SPSS (version 16). *p*-values ≤ 0.05 were considered significant.

## 3. Results and Discussion

### 3.1. Determination of the Tocotrienol, Tocopherol, and γ-Oryzanol Contents

**Figure 1:**
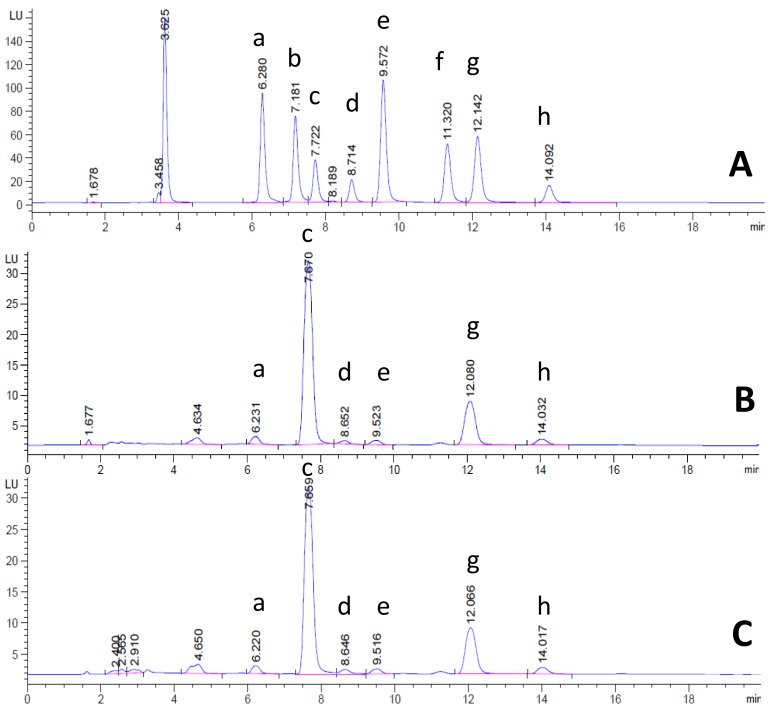
HPLC chromatogram of reference tocotrienols, tocopherols, and natural purple rice bran oils (NPRBOs). (**A**) Mixed reference tocotrienols and tocopherols, including δ-tocotrienol (a), β-tocotrienol (b), γ-tocotrienol (c), α-tocotrienol (d), δ-tocopherol (e), β-tocopherol (f), γ-tocopherol (g), and α-tocopherol; and NPRBOs including (**B**) Khao’ Gam Leum-Phua and (**C**) Khao’ Gam Pah E-Kaw.

**Figure 2:**
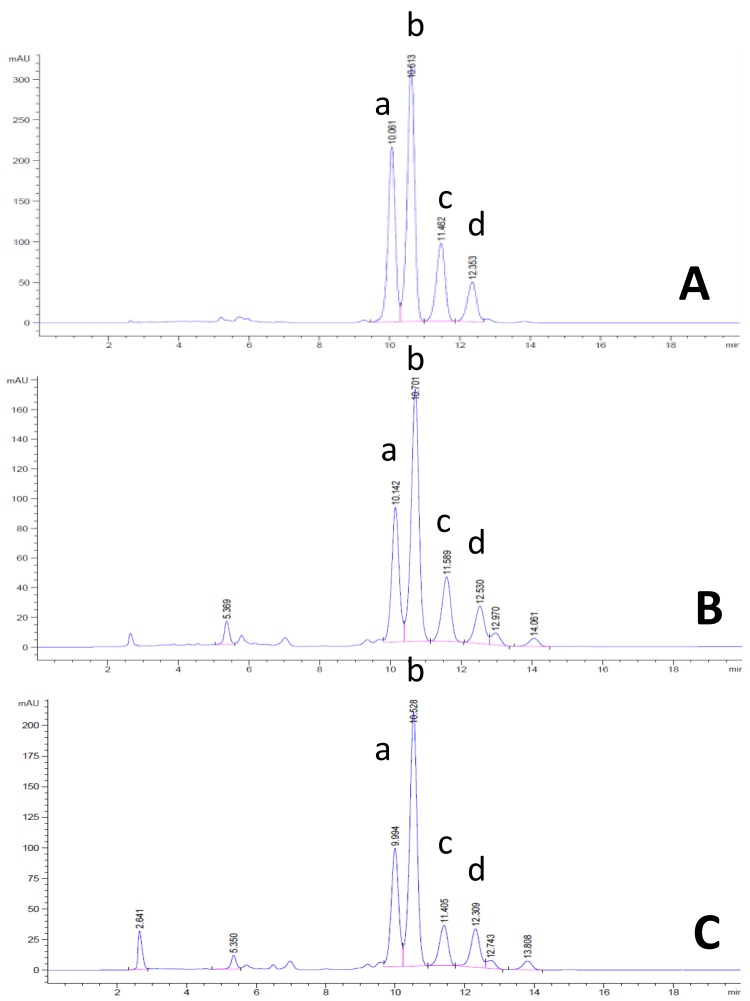
HPLC chromatogram of reference γ-oryzanol and NPRBOs. (**A**) γ-oryzanol, which is composed of cycloartenyl ferulate(a), 24-methylenecycloartanyl ferulate (b), campesteryl ferulate (c), and sitosteryl ferulate (d); and NPRBOs, including (**B**) Khao’ Gam Leum-Phua and (**C**) Khao’ Gam Pah E-Kaw.

In this study, it was found that the NRPBOs were comprised of δ-, γ-, and α-tocotrienol, δ-, γ-, and α-tocopherol, and γ-oryzanol, which corresponds to a previous report by Pengkumsri et al., who reported that Chiang Mai black rice bran oil prepared by supercritical carbon dioxide extraction is composed of δ-, γ-, and α-tocotrienol, δ-, γ-, and α-tocopherol, and γ-oryzanol, while Supanburi rice bran oil (white rice) is composed of δ- and γ-tocotrienol, δ- and γ-tocopherol, and γ-oryzanol [16]. The amounts of all components of NRPBOs are shown in Table 1, and their HPLC chromatograms are provided in Figure 1 (tocotrienols, tocopherols) and Figure 2 (γ-oryzanol). NPRBO prepared from Khao’ Gam Leum-Phua exhibited the highest δ- and γ-tocotrienol and δ-, γ-, and α-tocopherol contents, while NPRBO prepared from Khao’ Gam Pah E-Kaw showed the highest α-tocotrienol and γ-oryzanol contents. The amount of γ-oryzanol in the NPRBOs of Khao’ Gam Pah E-Kaw (KGPEK), Khao’ Gam Boung (KGB), and Khao’ Gam Thor (KGT) corresponded to the previous report by Saenjum et al. who prepared γ-oryzanol-rich extracts using the solvent extraction technique [10]. Interestingly, the amount of γ-oryzanol in the NPRBO in Khao’ Niaw Dam (KND) was higher than previously reported using this raw material. In the present study, KND was collected from the Mae Hong Son Rice Research Center, Mae Hong Son Province, while, in the previous report, KND was collected from the Phob-Pra Agricultural Extension Office, Tak Province.

### 3.2. Investigation of the Anti-Inflammatory Activity of NPRBO Via Nitric Oxide and COX-2 Inhibition

The anti-inflammatory- activity of the NPRBOs was determined through their inhibitory effects on nitric oxide and iNOS production in RAW 264.7 cells induced with LPS and IFN-γ as well as the inhibition of COX-2 production by HT-29 and HCT116 cells induced with LPS and IFN-γ. NPRBOs were effective in inhibiting the LPS/IFN-γ-mediated induction of nitric oxide, iNOS, and COX-2 production, which was comparable to the positive controls—curcumin and γ-oryzanol—as shown in Table 2. The NPRBO prepared from Khao’ Gam Leum-Phua exhibited the highest inhibitory activity on nitric oxide and iNOS production in RAW 264.7 cells and showed the highest inhibitory activity on COX-2 production in colorectal cell lines at different stages of colorectal cancer, i.e., HT-29 adenocarcinoma cells and HCT116 carcinoma cells. All NPRBOs inhibited nitric oxide and iNOS production by LPS/IFN-γ-stimulated RAW 264.7 cells, and COX-2 production from LPS/IFN-γ-stimulated HT-29 and HCT116 cells in a dose-dependent manner without the presence of cytotoxicity in RAW 264.7, HCT 116, and HT-29 cells at concentrations lower than 100 µg/mL. However, the inhibitory activities of all the NPRBOs were less than those of curcumin, a potent natural anti-inflammatory. Interestingly, the inhibitory activities of all the NPRBOs were higher than those of γ-oryzanol. It is proposed here that the potent anti-inflammatory property is mainly due to a combination of non-polar components, including tocotrienols, tocopherols, and γ-oryzanol. It is possible that tocotrienols, tocopherols, and γ-oryzanol may penetrate through the cell membrane due to their lipophilicity. These results correspond to previous studies that found that the tocotrienol-rich fraction of palm oil possesses potent anti-inflammatory activity through the inhibition of iNOS and COX-2 production, as well as NF-κB expression in human monocytic (THP-1) cells [24]. Moreover, black rice bran extracts have been shown to significantly suppress tetradecanoylphorbol acetate-induced inflammation and decrease the production of tumor necrosis factor- α (TNF-α), interleukin-1β (IL-1β), IL-6, and Leukotriene B4 (LTB4) [25]. Ju et al. reported that a γ-tocopherol-rich mixture of tocopherols (containing 57% γ-tocopherol, 24% δ-tocopherol, and 13% α-tocopherol) effectively inhibited colon carcinogenesis in azoxymethane/dextran sulfate sodium-treated mice. This inhibition was speculated to be due to the apoptosis-inducing, anti-inflammatory, antioxidant, and reactive nitrogen species-trapping activities of tocopherols [26]. Furthermore, γ-tocopherol is a more potent inhibitor than α-tocopherol in regard to neoplastic transformation during the post-initiation phase in 3-methylcholanthrene-treated C3H/10T1/2 murine fibroblasts, as reported by Cooney et al. [27]. Additionally, the Cooney group also found that γ­tocopherol could trap NO and other nitrogen free radicals more efficiently than α­tocopherol. This is interesting considering the high amount of γ­tocopherol in the NPRBOs and may be related to their inhibitory effects on nitric oxide and iNOS production.

The results from this study demonstrate that NPRBOs exhibited potent anti-inflammatory activity, especially regarding an inhibitory effect on COX-2 production in both colorectal cell lines (HT-29 and HCT116 cells). COX-2 plays a vital role in colorectal carcinogenesis and nonsteroidal anti-inflammatory drugs (NSAIDs), especially COX-2 selective inhibitors, have potential chemopreventive properties in human colorectal cancer [28]. Indeed, COX-2 inhibitors have been shown to inhibit cell proliferation, induce apoptosis, inhibit angiogenesis, and stimulate the immune system [29,30]. Shirode and Sylvester have shown that combined treatment with γ-tocotrienol and celecoxib, an inhibitor of cyclooxygenases, was able to reduce PGE_2_, COX-2, and phospho-Akt (active) levels in the highly malignant (_+_SA) mammary epithelial cell line. Moreover, combined treatment with γ-tocotrienol and celecoxib also downregulated NF-κB and induced apoptosis in mammary epithelial cells [31]. The present results indicate that NPRBO may possess potential anti-inflammatory activity. It is therefore likely that NPRBO containing tocotrienols, tocopherols, and γ-oryzanol may be a potential candidate for preventing colorectal carcinogenesis. NPRBO from KGLP was selected to prepare colorectal-targeted tablets based on its anti-inflammatory activity and tocotrienol, tocopherol, and γ-oryzanol content.

### 3.3. In Vitro Release Proof of Concept for the NPRBO Colorectal Delivery Platform 

The KGLP-NRPBO extract was in liquid form; thus, to formulate it in tablets, the NRPBO was absorbed using the absorbents Avicel^®^ PH 101 and Aerosil^®^. The ratios of NRPBO extract to the absorbents that provided an appropriate damp mass were 1:4 and 1:1 for Avicel ^®^ PH 101 and Aerosil^®^, respectively. Polyvinylpyrrolidone (PVP) was added to enhance the compression property of the KGLP-NRPBO tablets, and tableting excipients (talcum, Ac-Di-Sol^®^, and magnesium stearate) were added to NRPBO granules to enable the tableting process. Both KGLP-NRPBO: Avicel^®^ PH 101 1:4 and KGLP-NRPBO: Aerosil^®^ 1:1 tablets showed less than one percent weight loss in the friability test, which is suitable for the film coating process [32].

In the coating process, the peeled film-coated tablets were observed in the batch that used Avicel^®^ PH 101 as the absorbent. This phenomenon contributes to the poor absorption properties of Avicel^®^ PH 101 at the coating temperature. Consequently, the KGLP-NRPBO extract diffused from the core tablets to the films, and caused them to swell and peel off from the tablets. On the other hand, the core tablets that used Aerosil^®^ as the absorbent were smoothly coated film tablets. Therefore, they were used in the in vitro release assay in simulated gastrointestinal pH media.

**Figure 3:**
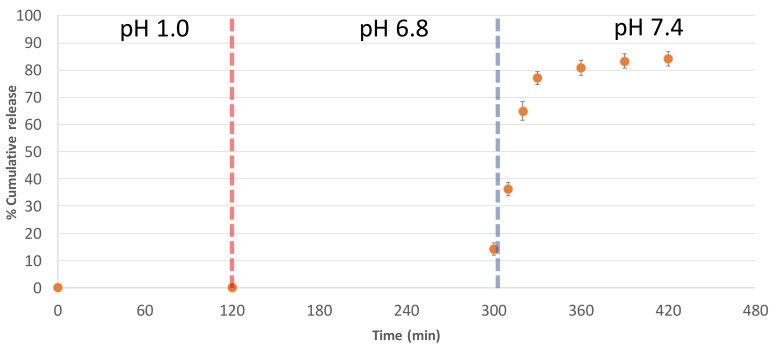
In vitro NPRBO release from film-coated tablets (*n* = 5) in three-phase simulated gastrointestinal pH media.

The simulated gastrointestinal study was divided into three phases: the gastric phase at pH 1, the small intestinal phase at pH 6.8, and the colorectal phase at pH 7.4. γ-Oryzanol was used as a marker for the in vitro release study. Figure 3 demonstrates the percentage of γ-oryzanol cumulative release in each phase. As expected, no γ-oryzanol was observed in the gastric phase, since Eudragit^®^ L-100 D was used as the outer coating of the KGLP-NPRBO tablets. Approximately 15% of γ-oryzanol was released in the small intestinal phase. This was a consequence of the Eudragit^®^ L-100 D outer coat that dissolved at pH 6.8, so that the Eudragit^®^ NE30D inner coating layer swelled but did not dissolve since it is a time-dependent controlled-release polymer. Thus, some γ-oryzanol diffused through the swelling layer of the Eudragit^®^ NE30D in a zero-order fashion, according to the γ-oryzanol gradient between the core tablet and the media. Premature release is a common problem that occurs with time-dependent controlled-release polymers [33]. In this case, the Eudragit^®^ NE30D film was not thick enough, so, as a consequence, some γ-oryzanol was released earlier in the small intestinal phase. However, up to 85% of the γ-oryzanol was released in the colorectal phase, as the Eudragit^®^ NE30D film dissolved entirely after 120 min. The lag-time of 5 h in the gastric and small intestinal phases was sufficient to deliver the KGLP-NPRBO tablets to the colorectal area, since the gastro-intestinal transit time is approximately 3–5 h, after which the bolus stays in the colon for the next 36–72 h [34,35]. Based on this result, it is speculated that, upon administrating the NPRBO film-coated tablets to the human body, the dietary delivery system of the KGLP-NPRBO tablets would be feasible for the delivery of γ-oryzanol to the targeted colorectal region.

## 4. Conclusions

It was found that the NRPBOs were comprised of δ-, γ-, and α-tocotrienol, δ-, γ-, and α-tocopherol, and γ-oryzanol. Khao’ Gam Leum-Phua exhibited the highest δ- and γ-tocotrienol and δ-, γ-, and α-tocopherol contents, while the NPRBO prepared from Khao’ Gam Pah E-Kaw showed the highest α-tocotrienol and γ-oryzanol contents.

The present results indicate that the NPRBOs exhibited potent anti-inflammatory activity in a cell-based study, comparable to the positive controls, curcumin and γ-oryzanol. The NPRBO prepared from Khao’ Gam Leum-Phua showed the highest inhibitory activity on nitric oxide and iNOS production in LPS/IFN-γ-stimulated RAW 264.7 cells and also exerted the highest inhibitory activity on COX-2 production in both colorectal cell lines, i.e., HT-29 adenocarcinoma cells and HCT116 carcinoma cells. It is suggested that there is a potential use for NPRBO containing tocotrienols, tocopherols, and γ-oryzanol as a colorectal chemopreventive agent.

Moreover, this study demonstrated that the NPRBO colorectal dietary supplement delivery concept is feasible by using Aerosil^®^ as an absorbent with KGLP-NPRBO to produce KGLP-NPRBO core tablets. Eudragit^®^ NE30D and Eudragit^®^ L-100 D were successfully used as coating materials for delivering NPRBO in simulated gastrointestinal pH media. The film-coated NPRBO tablets did not show premature release at gastric and duodenum pH. Eighty-five percent of γ-oryzanol was released after 6 h at pH 7.4; thus, the film-coated NPRBO tablets are expected to reach the colorectal area. This study demonstrates the potential anti-inflammatory activity (COX-2 inhibition) of KGLP-NPRBO which is one of the pathways that causes colorectal cancer and provides proof of concept for a colorectal-specific dietary supplement delivery system. However, further in vivo and clinical studies are required to explore the pharmacological significance and pharmacokinetics of NPRBO in colon cancer and local diseases.

## Figures and Tables

**Table 1:** Tocotrienol, tocopherol, and γ-oryzanol contents in NPRBOs.

Samples	Components (mg/g NPRBO)						
	δ-Tocotrienol	γ-Tocotrienol	α-Tocotrienol	δ-Tocopherol	γ-Tocopherol	α-Tocopherol	γ-Oryzanol
Khao’ Gam Boung	0.09 ± 0.02 ^b^	1.42 ± 0.08 ^a,b^	0.18 ± 0.02 ^b^	0.09 ± 0.02 ^b^	1.03 ± 0.08 ^a^	0.35 ± 0.03 ^c,d^	9.73 ± 0.47 ^a,b^
Khao’ Gam Thor	0.10 ± 0.02 ^a,b^	1.33 ± 0.09 ^b^	0.17 ± 0.02 ^b^	0.09 ± 0.02 ^b^	0.95 ± 0.07 ^b^	0.32 ± 0.03 ^d^	9.32 ± 0.52 ^b^
Khao’ Gam Leum-Phua	0.13 ± 0.03 ^a^	1.57 ± 0.10 ^a^	0.22 ± 0.03 ^a^	0.12 ± 0.02 ^a^	1.06 ± 0.08 ^a^	0.48 ± 0.03 ^a^	9.88 ± 0.58 ^a,b^
Khao’ Gam Pah E-Kaw	0.10 ± 0.02 ^a,b^	1.46 ± 0.09 ^a,b^	0.23 ± 0.03 ^a^	0.11 ± 0.03 ^a,b^	0.98 ± 0.07 ^a,b^	0.43 ± 0.04 ^a,b^	10.11 ± 0.48 ^a^
Khao’ Niaw Dam	0.09 ± 0.02 ^b^	1.44 ± 0.07 ^a,b^	0.20 ± 0.02 ^a,b^	0.10 ± 0.02 ^a,b^	1.00 ± 0.06 ^a,b^	0.38 ± 0.04 ^b,c^	9.68 ± 0.45 ^a,b^

Data are expressed as means ± SD; triplicates; *n* = 3 Mean values within a column superscripted by the same letter are not significantly different at *p* ≤ 0.05.

**Table 2:** Anti-inflammatory activities of NPRBOs.

Samples	50% Inhibition Concentration (µg/mL)			
	Nitric Oxide	iNOS	COX-2	
			HCT 116	HT-29
Khao’ Gam Boung	19.46 ± 1.19 ^d^	24.62 ± 1.55 ^b,c^	22.24 ± 1.22 ^b^	21.08 ± 0.84 ^c^
Khao’ Gam Thor	22.28 ± 1.12 ^e^	27.46 ± 1.68 ^c,d^	23.27 ± 0.64 ^b,c^	22.18 ± 0.72 ^c,d^
Khao’ Gam Leum-Phua	15.19 ± 0.75 ^b^	22.72 ± 1.47 ^b^	21.08 ± 0.87 ^b^	18.73 ± 0.76 ^b^
Khao’ Gam Pah E-Kaw	17.02 ± 0.84 ^b,c^	24.08 ± 1.32 ^b,c^	22.48 ± 0.63 ^b^	21.26 ± 0.82 ^c^
Khao’ Niaw Dam	18.67 ± 0.79 ^c,d^	25.25 ± 1.30 ^b,c^	27.39 ± 1.02 ^d^	26.75 ± 0.92 ^e^
Gamma-oryzanol	35.36 ± 1.56 ^f^	29.52 ± 1.42 ^d^	35.75 ± 1.28 ^e^	33.47 ± 0.86 ^f^
Curcumin	12.52 ± 0.63 ^a^	15.55 ± 1.34 ^a^	16.14 ± 0.65 ^a^	14.29 ± 0.58 ^a^

Data are expressed as means ± SD; duplicates; *n* = 3 Mean values within a column superscripted by the same letter are not significantly different at *p* ≤ 0.05.
